# Thymic MALT lymphoma associated with Sjögren’s syndrome with postoperative cardiac tamponade and acute pleuritis: a case report

**DOI:** 10.1186/s12957-024-03442-1

**Published:** 2024-06-20

**Authors:** Takao Shigenobu, Takahiro Suzuki, Hiroyuki Hayashi, Akira Yoshizu

**Affiliations:** 1 Department of Thoracic Surgery, Yokohama Municipal Citizen’s Hospital, 1-1, Mitsuzawanishimachi, Kanagawa-ku, Yokohama, 221-0855 Japan; 2 Department of Pathology, Yokohama Municipal Citizen’s Hospital, 1-1, Mitsuzawanishimachi, Kanagawa-ku, Yokohama, 221-0855 Japan

**Keywords:** Cardiac tamponade, Pleuritis, Serositis, Sjogren’s syndrome, Thymic MALT lymphoma

## Abstract

**Background:**

Thymic mucosa-associated lymphoid tissue (MALT) lymphoma is rare and is known to be associated with Sjögren’s syndrome (SjS). SjS is rarely accompanied by serositis. Here, we describe the first case of postoperative cardiac tamponade and acute pleuritis in a patient with thymic MALT lymphoma associated with SjS.

**Case presentation:**

A 33-year-old woman with SjS presented with an anterior mediastinal mass on chest computed tomography, which was performed for further examination of the condition. Suspecting a thymic MALT lymphoma or thymic epithelial tumor, total thymectomy was performed. The mediastinal mass was histopathologically diagnosed as a thymic MALT lymphoma. The patient was discharged with a good postoperative course but visited the hospital 30 days after surgery for dyspnea. Cardiac tamponade was observed and drainage was performed. Four days after pericardial drainage, chest radiography revealed massive left pleural effusion, and thoracic drainage was performed. The patient was diagnosed with serositis associated with SjS and treated with methylprednisolone, which relieved cardiac tamponade and pleuritis.

**Conclusions:**

Surgical invasion of thymic MALT lymphomas associated with SjS may cause serositis. Postoperative follow-up should be conducted, considering the possibility of cardiac tamponade or acute pleuritis due to serositis as postoperative complications.

## Background

Thymic mucosa-associated lymphoid tissue (MALT) lymphoma is a rare low-grade extranodal marginal zone B-cell lymphoma [1]. Although the disease is extremely rare, most reports indicate that if resectable, surgical resection is performed as the initial treatment with a favorable postoperative prognosis [2]. Thymic MALT lymphoma is reported to be more common in middle-aged Asian women and is associated with autoimmune diseases, especially Sjögren’s syndrome (SjS) [2–4]. Among autoimmune diseases, rheumatoid arthritis and systemic lupus erythematosus are well known to be associated with serositis; however, SjS has rarely been reported to be associated with pericarditis and pleuritis [5, 6]. In this report, we describe, for the first time, a case of dyspnea due to cardiac tamponade and acute pleuritis one month after surgery for thymic MALT lymphoma associated with SjS, which was treated with pericardial drainage, thoracic drainage, and methylprednisolone.

## Case presentation

**Fig. 1:**
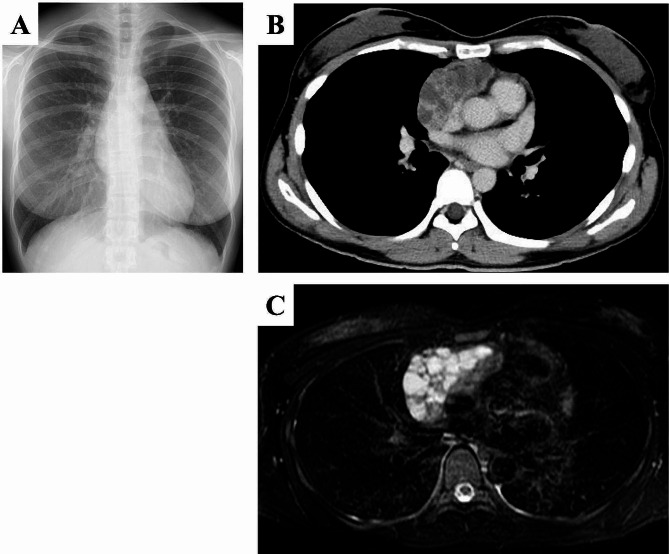
Imaging findings of thymic mucosa-associated lymphoid tissue. A chest radiograph shows a tumor with a positive silhouette sign in the right atrium. There is no evidence of cardiomegaly (**A**). Chest computed tomography shows a 9.0 cm mass consisting of a heterogeneous multifocal cyst and a contrast-effective septate in the anterior mediastinum (**B**). T2-weighted magnetic resonance imaging of the chest shows a lobulated mass with cyst-like high-signal clusters. The tumor does not invade the surrounding organs (**C**)

A 33-year-old woman with asthma and atopic dermatitis visited a previous hospital with the chief complaint of dry mouth and eyes that had persisted for more than three months. The patient had no history of medication use or smoking, and no family history of autoimmune disease; however, her grandmother had tested positive for rheumatoid factor. Laboratory data revealed leukopenia (2630/µL). Screening test for autoimmune disease revealed positive anti-nuclear antibodies with a titer of 1:1280, elevated serum rheumatoid factor to ≥ 500 IU/mL, elevated anti-SS-A antibodies to ≥ 1200 U/mL, and elevated anti-SS-B antibodies to 17.3 U/mL. Anti-double-stranded DNA antibody, anti-Sm antibody, anti-cardiolipin antibody, and lupus anticoagulant test results were negative. Additionally, hyperproteinemia (total protein, 9.8 g/dL) and hypergammaglobulinemia (IgG, 3529 mg/dl; IgA, 1779 mg/dl) were observed. The serum soluble interleukin-2 receptor level was within normal limits (363 U/mL). Schirmer’s test revealed 1 mm/5 min in the left eye and 6 mm/5 min in the right eye. The patient fulfilled the American College of Rheumatology and the European League Against Rheumatism Classification Criteria for primary SjS and was diagnosed with SjS [7]. Chest radiography and contrast-enhanced computed tomography (CT) showed an anterior mediastinal mass with multiple cysts and an enhancing effect on the septal wall (Fig. 1A, B). Magnetic resonance imaging showed a lobulated mass with cyst-like high-signal clusters on T2-weighted images (Fig. 1 C). ^18^F-fluorodeoxyglucose positron emission tomography revealed a standardized uptake value of 5.0 in the anterior mediastinal mass, with no metastatic findings.

The diagnosis of SjS and imaging findings suggested thymic MALT lymphoma or cystic thymoma. The lesion was considered resectable, and a total thymectomy was performed through a median sternotomy. The tumor was easily removed with no pericardial or pleural damage.

**Fig. 2:**
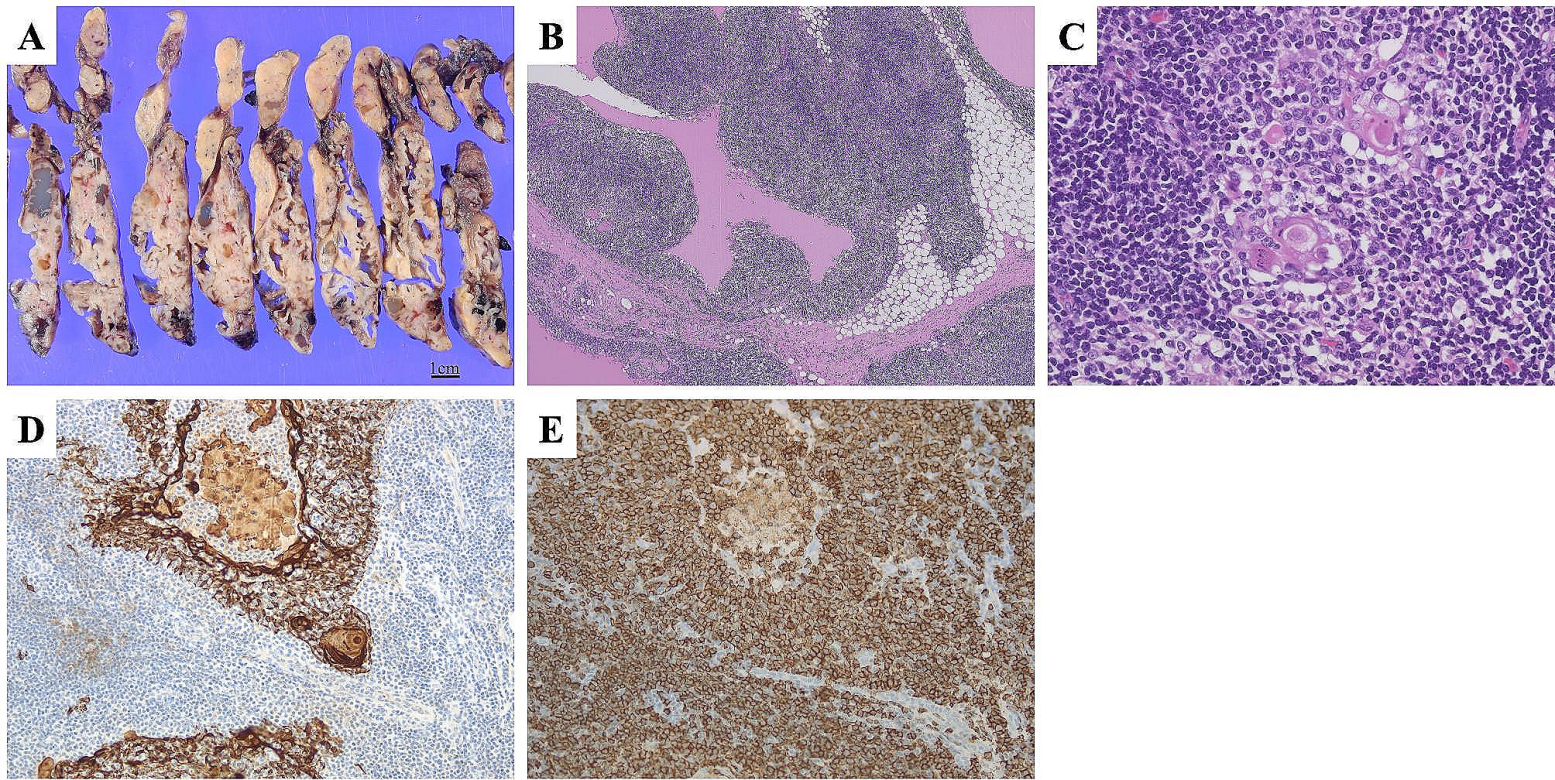
Histopathological examination of thymus. The resected tumor is well circumscribed and consists of fleshy, white-colored tissue and is interspersed with multiple variably sized cysts (**A**). A sizeable cystic lesion containing eosinophilic fluid is lined by thymic epithelium and lymphocytes. The pale areas consist of thymic epithelium infiltrated by lymphocytes (**B**, hematoxylin and eosin stain, x40**)**. A Hassall corpuscle surrounded by lymphocytes (**C**, hematoxylin and eosin stain, x200**)**. Immunostaining of cytokeratin AE1/AE3 highlights lymphoepithelial lesions (**D**, x200**)** and a dense infiltrate of CD20-positive cells around a Hassall’s corpuscle (**E**, x200**)**

The resected specimen was a 12.0 × 10.0 × 2.0 cm mass with a smooth surface and multiple variably sized cysts (Fig. 2A). The cystic septum was white, and serous fluid and blood accumulated in the cyst. Histologically, the cyst wall was covered with stratified squamous epithelium, with dense infiltration of lymphocytes and the formation of lymphoepithelial lesions (Fig. 2B, C). Immunostaining showed that the lymphocytes infiltrating the epithelium were positive for CD20 (Fig. 2D, E). Based on these findings, thymic MALT lymphoma was diagnosed.

**Fig. 3:**
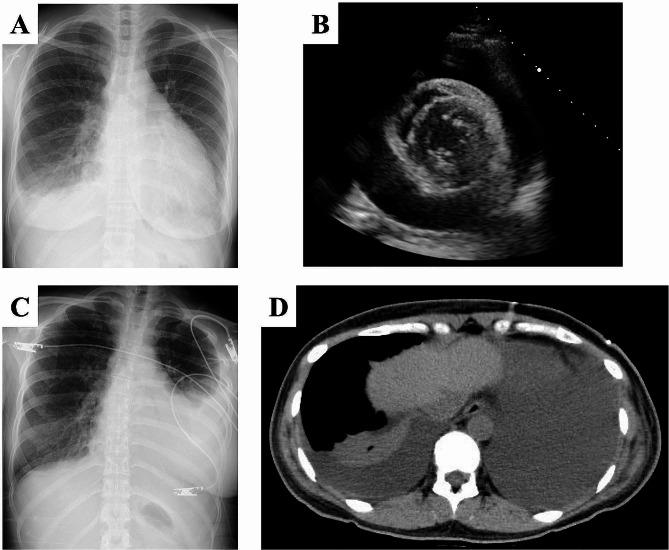
Imaging findings of cardiac tamponade and acute pleuritis after thymectomy. Chest radiograph shows obvious cardiomegaly with a 62% cardio-thoracic ratio (**A**), and an echocardiographic parasternal short-axis view shows massive pericardial effusion (**B**). After pericardial drainage, chest radiograph shows pleural effusion in the left thoracic cavity (**C**), and chest computed tomography shows bilateral pleural effusions, especially on the left, with massive pleural effusion and the heart shifted to the right. Pericardial effusion is hardly observed (**D**)

The postoperative course was uneventful, and the patient was discharged on the seventh postoperative day. However, on the 30th postoperative day, the patient visited the hospital with gradually worsening dyspnea. Physical examination revealed no fever, but respiratory distress and jugular venous distension. Chest radiography revealed an enlarged cardiac silhouette (Fig. 3A), and echocardiography revealed massive pericardial effusion (Fig. 3B), dyspnea due to cardiac tamponade was diagnosed, and pericardial drainage was performed after emergency hospitalization. After draining 600 mL of pericardial fluid, the drain was removed on the fourth day of hospitalization. However, chest radiography and CT on the same day showed massive pleural effusion on the left side (Fig. 3C, D), and thoracic drainage was performed. Both pericardial and pleural effusions were exudative. Laboratory examination of the exudate revealed predominantly mononuclear cells with normal sugar levels (64 and 126 g/dL, respectively), almost normal adenosine deaminase levels (22.6 and 23.3 U/L, respectively), and negative bacterial culture tests, including acid-fast bacteria. Cytological examination revealed no malignant findings, mostly consisting of mesothelial cells and small lymphocytes without atypia. The patient was diagnosed with serositis associated with SjS and treatment was initiated with methylprednisolone (32 mg/day). On the first day after thoracic drainage, the outflow was 830 mL/day; however, by the fourth day, it decreased to 35 mL/day. The thoracic drain was removed on the fifth day, and the patient was discharged on the 15th day of hospitalization. At present, 30 months after surgery, methylprednisolone has been tapered to 2 mg/day, and neither thymic MALT lymphoma nor serositis has recurred.

## Discussion and conclusions

Thymic MALT lymphoma is rare and accounts for approximately 1% of all anterior mediastinal tumors [8]. Approximately 80% of thymic MALT lymphoma cases are associated with autoimmune disorders, most of which are SjS [2, 3, 9]. There are a few scattered surgical case reports and case series on thymic MALT lymphoma, but none have described cardiac tamponade or acute pleuritis as postoperative complications.

Pericardial involvement in systemic autoimmune diseases is often asymptomatic, with pericardial effusions commonly seen in rheumatoid arthritis, systemic lupus erythematosus, and systemic sclerosis but rarely described in SjS [10]. In a review of cardiac manifestations in 31 patients with SjS, pericardial effusion and pericarditis were described in 19% and 3% of patients, respectively, and were reported to improve with treatment with steroids or non-steroidal anti-inflammatory drugs [6]. Three cases of cardiac tamponade associated with SjS have been reported, including one case of secondary SjS associated with systemic sclerosis, all of which improved rapidly after treatment with steroids [11–13]. Of these, only one case had pericardial drainage for pericardial effusion associated with SjS. Although there is no cell fractionation or laboratory data description, it has been described as an exudative effusion [11].

Pleural involvement in systemic autoimmune diseases is commonly seen in rheumatoid arthritis and systemic lupus erythematosus, but is rare in SjS, with a reported frequency of less than 1% [5]. It has been described that the pleural effusions in patients with SjS are lymphocyte-predominant and exudative, with normal glucose and low adenosine deaminase levels. In addition, high levels of anti-SS-A/anti-SS-B-antibody and low complement levels have been reported [5, 14]. Almost all cases of pleuritis associated with SjS described to date have been treated with steroids and have shown improvement, although a few cases of improvement without treatment have been reported [15–18].

Although the etiology of serositis associated with SjS remains unclear, analysis of pleural effusion has demonstrated the occurrence of local immune reactions in the pleura, including an increase in lymphocytes, production of rheumatoid factor and anti-SS-A/SS-B antibodies, formation of immune complexes, and complement activation [19, 20]. In the present case, we did not immunologically evaluate the pericardial and pleural effusions and were unable to directly assess the relationship between SjS and serositis.

In the present case, the patient had acute-onset serositis, which is rare during the clinical course of SjS. The cause was thought to be that the patient had untreated SjS and was prone to serositis because of surgical invasion and inflammation, which led to acute pericarditis due to surgery and acute pleuritis due to pericardial drainage. Although it is difficult to prove whether SjS causes serositis or demonstrate the mechanism of serositis development, Hosoda et al. reported that a rapid response to steroids could help diagnose pleuritis associated with SjS based on previous reports [16]. In this case, after initiating methylprednisolone, serositis improved immediately, suggesting that SjS contributed to the postoperative complications of cardiac tamponade and acute pleuritis.

In the present case, surgery and pericardial drainage induced cardiac tamponade and acute pleuritis in a patient with thymic MALT lymphoma and SjS. Postoperative follow-up of patients with thymic MALT lymphoma with SjS should be considered as a possible postoperative complication of acute serositis.

## Data Availability

No datasets were generated or analysed during the current study.

## Abbreviations

CT: Computed tomography
MALT: Mucosa-associated lymphoid tissue
SjS: Sjögren’s syndrome
